# Machine learning-based prediction of herbal medicine response in functional dyspepsia: protocol for a randomized, assessor-blinded, multicenter trial

**DOI:** 10.3389/fmed.2026.1716891

**Published:** 2026-02-11

**Authors:** Chaehyun Park, Hayun Jin, Boram Lee, Young-Eun Choi, Ojin Kwon, Mi Young Lim, Donghyun Nam, Dong-Jun Choi, Jun-Hwan Lee, Jae-Woo Park, Seok-Jae Ko, Hojun Kim

**Affiliations:** 1 Department of Korean Internal Medicine, College of Korean Medicine, Kyung Hee University, Kyung Hee University Hospital at Gangdong, Seoul, Republic of Korea; 2 KM Science Research Division, Korea Institute of Oriental Medicine, Daejeon, Republic of Korea; 3 Clinical Research Coordinating Team, Korea Institute of Oriental Medicine, Daejeon, Republic of Korea; 4 Personalized Diet Research Group, Food Functionality Research Division, Korea Food Research Institute, Wanju, Republic of Korea; 5 Department of Diagnostics and Biofunctional Medicine, College of Korean Medicine, Sangji University, Wonju, Republic of Korea; 6 Department of Internal Korean Medicine, Dongguk University Ilsan Oriental Medical Hospital, Goyang, Republic of Korea; 7 Korean Medicine Life Science, University of Science and Technology, Campus of Korea Institute of Oriental Medicine, Daejeon, Republic of Korea; 8 Department of Digestive Diseases, College of Korean Medicine, Kyung Hee University, Seoul, Republic of Korea; 9 Department of Rehabilitation Medicine of Korean Medicine, College of Korean Medicine, Dongguk University, Gyeongju, Republic of Korea

**Keywords:** functional dyspepsia, herbal medicine, machine learning, personalized medicine, prediction algorithm, randomized clinical trial

## Abstract

**Background:**

The purpose of this study is to evaluate the predictive accuracy of a machine learning-based herbal medicine response prediction algorithm in patients with functional dyspepsia (FD). In a preliminary clinical study, the algorithm was developed using the XGBoost regressor framework to predict the relative effect sizes of three commonly prescribed herbal formulations—*Yijung-tang* (*Lizhong-tang*), *Pyeongwi-san* (*Pingwei-san*), and *Shihosogan-tang* (*Chaihu Shugan-tang*). The prediction system recommends the formulation expected to yield the greatest therapeutic benefit for each individual.

**Methods:**

This study is a randomized, assessor-blinded, parallel-group, open-label, multicenter clinical trial. A total of 100 patients with FD will be recruited from two Korean medical hospitals and randomly assigned to either the ACCORD group (*n* = 50), which will receive treatment guided by the machine learning algorithm, or the DISCORD group (*n* = 50), which will receive one of the two treatments not recommended by the algorithm. Patients will take the assigned herbal medicine for 8 weeks, three times daily, between meals.

**Outcomes:**

The primary outcome will be gastrointestinal symptom score. Secondary outcomes will include total dyspepsia symptom score, adequate relief of dyspepsia, overall treatment effect, visual analog scale score, functional dyspepsia–related quality of life, and pattern identification questionnaire results. Exploratory outcomes will include blood and fecal metabolome analysis, fecal and salivary microbiota profiling, and measurements obtained using Korean medicine diagnostic devices (heart rate variability, tongue, pulse, and abdominal diagnosis).

**Conclusion:**

Integrating a machine learning-based prediction system into treatment strategies for FD may enhance clinical practice and support the broader adoption of artificial intelligence-driven approaches in personalized medicine.

**Clinical trial registration:**

Clinical Research information Service (registration number: KCT0010587) and Open Science Framework (https://osf.io/2ecz8).

## 1 Introduction

Functional dyspepsia (FD) is a medical condition characterized by one or more of the following symptoms: postprandial fullness, early satiation, epigastric pain, and epigastric burning, which remain unexplained after routine clinical evaluation. FD is classified into two subcategories: postprandial distress syndrome (PDS), characterized by meal-induced dyspeptic symptoms, and epigastric pain syndrome (EPS), which does not occur exclusively postprandially; the two subgroups may also overlap. The pathophysiology of FD is complex, multifactorial, and not fully elucidated (1). The global prevalence of FD is estimated at 21%, although rates vary substantially between countries (2).

Conventional treatments for FD focus on symptom management, primarily through medication, with treatment durations typically limited to 8–12 weeks due to the absence of curative options (3). Acid suppressants, such as proton pump inhibitors, are mainly used for patients with EPS, whereas prokinetics are primarily prescribed for PDS with gastrointestinal motility issues. If these medications are ineffective, fundus-relaxing drugs may be used for PDS, and further treatment options include antidepressants or psychotherapy (4). However, these treatments are not universally effective, and most act by blocking specific neurotransmitter receptors, which can cause side effects such as extrapyramidal symptoms (5), QT interval prolongation (6), dizziness (7), and loss of appetite (8).

To address these limitations, traditional Korean medicine offers alternative treatments, such as herbal medicine and acupuncture, which are actively practiced worldwide (9). In Korea, clinical practice guidelines for FD in traditional Korean medicine have been developed, recommending herbal treatments for symptom improvement and maintenance. Notable prescriptions include *Banhasasim-tang*, *Yukgunja-tang*, *Soyo-san*, *Pyeongwi-san*, and *Shihosogan-san* (10). Traditional Korean medicine categorizes FD into six syndromic patterns: spleen and stomach deficiency–cold, spleen and stomach damp–heat, tangled cold and heat, spleen deficiency with qi stagnation, liver–stomach disharmony, and food retention disorder (11). In a study by Ha et al. (11), 95 patients with FD were analyzed, revealing that spleen and stomach deficiency–cold was the most prevalent pattern, accounting for 52% of cases, followed by spleen and stomach damp–heat (14.7%), tangled cold and heat (13.7%), spleen deficiency with qi stagnation (9.5%), liver–stomach disharmony (7.4%), and food retention disorder (3.2%).

Diagnosis in traditional Korean medicine is conducted through four phases: inspection, listening and smelling, inquiry, and palpation. Practitioners integrate correlations between the symptoms and signs presented by the patient to perform pattern identification, which guides the selection of appropriate treatments (12). Although recent advancements have led to the development of standardized questionnaires for quantitative assessments of specific patterns—such as spleen and stomach deficiency–cold, spleen and stomach damp–heat, and food stagnation in patients with FD—the diagnostic protocols in clinical practice remain heavily reliant on traditional methods, resulting in a process that is subjective and lacks standardization (13). To address these challenges, the integration of modern science with traditional Korean medicine, particularly through artificial intelligence (AI), has been proposed as a promising approach. AI has the potential to analyze data-driven insights and predict herbal medicine responses, thereby enabling more personalized and effective treatment strategies (14).

Empirically, herbal formulations such as *Yijung-tang* (*Lizhong-tang*, YJT) for spleen deficiency (15, 16), *Pyeongwi-san* (*Pingwei-san*, PWS) for food stagnation (17), and *Shihosogan-tang* (*Chaihu Shugan-tang*, SST) for liver depression (18) are widely used in the treatment of FD. In a preliminary study, these formulations were administered to patients with FD to develop an machine learning-based predictive algorithm for anticipating herbal medicine responses (19). Over an 8-week treatment period, data were collected from various sources, including blood, fecal, and saliva samples, as well as questionnaires and traditional diagnostic devices. This comprehensive dataset was used to construct the machine learning-based prediction system, which was designed to propose typical pattern differentiation types. Building on this foundational work, the present study aims to validate the predictive accuracy of the machine learning-based prediction algorithm. By randomly assigning patients with FD to receive YJT, PWS, or SST based on the machine learning prediction—or one of the remaining herbal medicines not determined by the algorithm—the study seeks to enhance the objectivity and reproducibility of traditional Korean medicine treatments. This approach may provide a pathway toward more personalized and effective patient outcomes.

## 2 Methods

### 2.1 Study design and setting

#### 2.1.1 Study design and allocation

This study is a randomized, assessor-blinded, parallel-group, open-label, multicenter clinical trial. It will be conducted at two Korean medical hospitals: Kyung Hee University Hospital at Gangdong and Dongguk University Ilsan Oriental Medical Hospital. Participants who voluntarily sign the informed consent form will undergo screening examinations during their initial visit (screening visit), including interviews, vital sign measurements, questionnaire surveys, assessments with Korean medicine diagnostic devices, and laboratory tests. Eligible participants will then be enrolled. Within 2 weeks of the screening visit, participants will be randomly assigned to either the ACCORD group, which will receive YJT, PWS, or SST based on a machine learning–driven herbal response prediction algorithm, or the DISCORD group, which will be randomly assigned one of the two herbal medicines not selected by the prediction algorithm from the three options. This ACCORD vs. DISCORD design is specifically employed to validate the algorithm’s discriminative ability—that is, whether the algorithm can accurately distinguish the optimal treatment from suboptimal alternatives for an individual patient. This validation of predictive accuracy is a necessary step before evaluating clinical utility against standard care.

The machine learning algorithm employed in this trial was developed in a prior preliminary clinical study (*N* = 265) (19) (the resulting paper detailing the development methodology is currently under review; Supplementary material 1). The algorithm utilizes the XGBoost (Extreme Gradient Boosting) regressor framework. The objective of the model is a regression task: to predict the magnitude of symptom improvement (effect size) for each of the three herbal formulations (YJT, PWS, and SST). The prediction system recommends the formulation expected to yield the greatest therapeutic benefit for the individual.

The ground truth (target variable) for model training was the observed change in the Gastrointestinal Symptom (GIS) score from baseline to 8 weeks in the preliminary study. The models were trained using comprehensive baseline data as input features. These features included demographics (e.g., age, sex, and FD duration); patient-reported outcomes and questionnaires (e.g., GIS, and various pattern identification questionnaires); data from Korean medicine diagnostic devices (e.g., tongue diagnosis system, radial pulse analyzer, and digital algometer readings); and laboratory test results (e.g., blood chemistry).

During development, the dataset was split into training (80%) and test (20%) sets for internal validation. Hyperparameter tuning was conducted using RandomizedSearchCV with 5-fold cross-validation on the training data. To mitigate overfitting due to high data dimensionality, an iterative feature selection process was employed to enhance model robustness. The final models demonstrated robust predictive accuracy on the independent test set, confirming their capability to predict treatment responses and discriminate the relative effectiveness among the herbal formulations. Detailed methodology of the algorithm development is provided in Supplementary material 1. The algorithm for prediction system employed in this trial is a locked version (v1.1) that will not be updated during the study period. The prospective validation of this locked algorithm in a new cohort is essential for evaluating its generalizability and mitigating potential biases or overfitting inherent in the preliminary training dataset.

**Figure 1:**
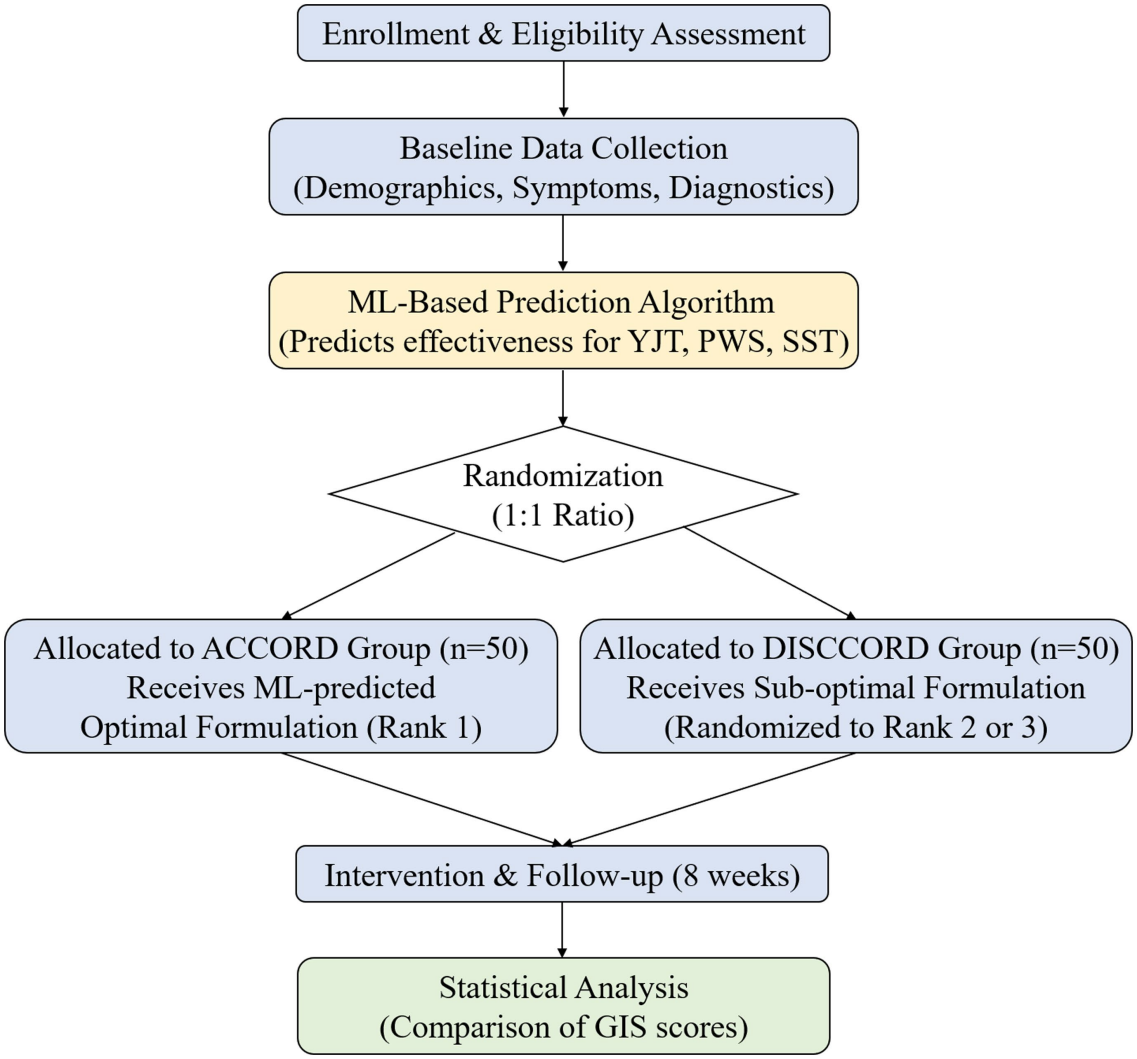
Flowchart of the trial intervention. ML, machine learning; YJT, *Yijung-tang*; PWS, *Pyeongwi-san*; SST, *Shihosogan-tang*; GIS, gastrointestinal symptom.

All groups will take the assigned clinical trial medications for 8 weeks. Participants will attend clinic visits at 4-week intervals until week 8 for evaluation of clinical variables, including vital signs, questionnaire surveys, and assessments with Korean medicine diagnostic devices (heart rate variability [HRV], tongue diagnosis, pulse diagnosis, and abdominal diagnosis). In addition, telephone visits will be conducted at 2-week intervals (weeks 2 and 6) to assess whether FD-related pain or discomfort has moderately improved since the previous treatment (Figure 1). Before and after taking the herbal medicine, blood samples (at screening and week 8) and stool and saliva samples (at weeks 0 and 8) will be collected to analyze blood and fecal metabolites as well as gut and oral microbiomes. A detailed assessment schedule is provided in Table 1.

**Table 1:** Clinical study schedule.

Assessment	Visit 1 (screening)	Visit 2 (baseline, week 0)	Visit 3 (week 2)	Visit 4 (week 4)	Visit 5 (week 6)	Visit 6 (week 8)
Written consent						
Demographic/medical history survey	●					
Physical measurements	●					
Vital sign measurements	●	●		●		●
Blood test	●					
Confirmation of inclusion / exclusion criteria	●					●
Random allocation		●				
Concomitant medications and adverse reaction confirmation		●	●	●	●	●
Gastrointestinal Symptom Score	●			●		●
Total Dyspepsia Symptom Score	●			●		●
Adequate relief of dyspepsia			●	●	●	●
Overall treatment effect				●		●
Dyspepsia Visual Analog Scale	●			●		●
Functional dyspepsia–related quality of life	●			●		●
Spleen Qi Deficiency Questionnaire	●			●		●
Stomach Qi Deficiency Questionnaire	●			●		●
Food Retention Questionnaire	●			●		●
Cold–Heat Pattern Questionnaire	●			●		●
Deficiency–Excess Pattern Questionnaire	●			●		●
Standard Tool for Pattern Identification of FD	●					
Korean medicine diagnosis (interview, tongue, pulse, abdominal)	●			●		●
Clinical trial medication prescription and administration education		●		●		
Clinical trial medication returns and compliance check				●		●
Stool kit and meal record sheet provision	●			●		
Stool sample collection, meal record sheet confirmation, and saliva sample collection		●				●
Confirmation of clinical study completion						●

#### 2.1.2 Randomization and blinding

Participants will be divided into the ACCORD group, assigned herbal medication by the machine learning-based prediction algorithm, and the DISCORD group, which will not use the prediction algorithm. For example, if the prediction algorithm assigns YJT, the group receiving YJT will constitute the ACCORD group, whereas those receiving PWS or SST will constitute the DISCORD groups. Randomization will be stratified by each herbal medicine using block randomization to minimize bias before the clinical trial begins. For each stratification factor, the group allocation ratio will be 1:1 (ACCORD: DISCORD), with block sizes of 4 and 8. The DISCORD group will then be divided into two subgroups, excluding the optimal herbal medicine per the prediction algorithm, and randomly assigned, resulting in a 2:1:1 allocation (ACCORD: DISCORD_1: DISCORD_2). To prevent bias toward a specific herbal medicine, the maximum number of participants assigned to any single herbal medicine will be capped at 48. Any additional participants for that medicine will be excluded during screening.

Randomization tables will be generated using SAS® Version 9.4 (SAS Institute Inc., Cary, NC, United States) by a medical statistician independent of the trial interventions and assessments. The randomization list will allow for the registration of 50 participants per institution. The randomization system will be managed by a medical statistician, independent of the trial, serving as the system administrator. Investigators will be able to access participant identification codes and group allocation information only with administrator approval. To ensure allocation concealment, a centralized, web-based randomization system will be utilized. This system will be managed by the independent medical statistician serving as the administrator. Once a participant is confirmed eligible and enrolled, the investigator will access the web-based system, which will then reveal the participant’s unique identification code and group allocation. The system ensures that the allocation sequence remains concealed until the point of assignment. Evaluation of participant outcomes will be conducted by staff who will not prescribe medications or access medical records and case report forms. Evaluators will ask only the questions necessary for assessment and will remain blinded to participant group assignments. These procedures, including centralized randomization managed by an independent statistician, allocation concealment, and assessor blinding, are implemented to minimize potential selection and assessment bias throughout the trial.

### 2.2 Eligibility criteria

#### 2.2.1 Inclusion criteria

• Adult men and women aged 19 years or older and 75 years or younger.
• Diagnosis of FD according to the Rome IV criteria:
 o Presence of at least one symptom among postprandial fullness, early satiation, epigastric pain, or epigastric burning.
 o Symptoms must have developed at least 6 months before screening and persisted for the past 3 months without organic findings.
• Dyspepsia severity of 40 points or higher on a 0–100 mm visual analog scale (VAS).
• Willingness to refrain from initiating any new treatments related to dyspepsia during the clinical trial period.
• Signed written informed consent, approved by the Institutional Review Board (IRB), after receiving a thorough explanation and voluntarily agreeing to participate in the study.

#### 2.2.2 Exclusion criteria

- Diagnosis of organic diseases such as peptic ulcers, esophageal cancer, MALT lymphoma, gastric cancer, colorectal cancer, or biliopancreatic diseases (asymptomatic gallstones are exempted) within 1 year prior to screening.
- Symptoms of gastroesophageal reflux disease or irritable bowel syndrome that are more severe than dyspepsia symptoms.
- Presence of alarm symptoms, including severe weight loss, hematochezia, dysphagia, odynophagia, or persistent vomiting.
- Significant neurological or psychiatric history or current conditions, including major depressive disorder, anxiety disorder, schizophrenia, anorexia nervosa, or epilepsy.
- Serious organic diseases (e.g., heart failure, angina, myocardial infarction, arrhythmia, valvular disease, malignant tumors, or stroke).
- History of gastrointestinal surgery (appendectomy permitted if performed more than 6 months prior to trial participation).
- Use of medications affecting the gastrointestinal tract, such as proton pump inhibitors, histamine receptor antagonists, antacids, gastrointestinal motility agents, fundus relaxants, mucosal protective agents, anticholinergics, adsorbents, antiflatulents, antiemetics, emetics, gastrin receptor antagonists, prostaglandin analogs, or *Helicobacter pylori* eradication therapy within 2 weeks prior to screening.
- Receipt of Korean traditional treatments such as acupuncture, herbal medicine, or moxibustion for dyspepsia within 2 weeks prior to screening or plans to receive such treatments during the study period.
- Use of antibiotics within 3 months prior to screening.
- Use of probiotic supplements within 1 month prior to screening (participation allowed if probiotics have been taken continuously for more than 1 month prior to screening without planned dosage changes during the trial).
- Severe liver or kidney dysfunction (as defined by AST or ALT levels more than three times the upper limit of normal, or serum creatinine levels more than twice the upper limit of normal at screening).
- History of significant alcohol or substance abuse.
- Pregnant or breastfeeding women, or women of childbearing potential unwilling to use effective contraception during the trial period.
- Conditions such as galactose intolerance, Lapp lactase deficiency, or glucose–galactose malabsorption.
- Known hypersensitivity to investigational medicinal products.
- Use of other investigational medicinal products within 3 months prior to the study.
- Chronic diseases not well controlled despite appropriate treatment (posing life-threatening risks, requiring hospitalization, causing severe physical disability, or substantially affecting daily life due to conditions such as hypertension or diabetes).
- Determined unsuitable for trial participation by the investigator or trial director for any other reason.

### 2.3 Participant recruitment

Participants will be recruited through regular promotional activities in mass media channels, including newspaper inserts and daily newspapers. Promotional materials will also be posted on bulletin boards and websites both within and outside the participating institutions. Regional advertisements such as subway and bus ads, as well as apartment bulletin board postings, will be used. Online platforms, including websites and applications, will additionally be utilized for advertising. Cooperation will further be sought from online communities, local gatherings, and clubs frequented by individuals with FD to generate interest and encourage participation.

### 2.4 Interventions

#### 2.4.1 Information and management of investigational medicinal products

The herbal medicine formulations used in this study are YJT, PWS, and SST, all of which are brown powders manufactured by HanKook ShinYak Co., Ltd. and Kyungbang ShinYak Co., Ltd. These products are approved for use under Korean medicine health insurance and are classified as gastrointestinal drugs by the Ministry of Food and Drug Safety (MFDS). They are administered orally at a recommended dosage of one packet three times daily between meals and should be stored in airtight containers at room temperature (1–30 °C). Each product has a shelf life of 36 months from the manufacturing date. The detailed composition of each herbal product is summarized in Table 2. These formulations are intended to support gastrointestinal health and are covered by Korean health insurance. Their production adheres to MFDS standards, ensuring quality and efficacy.

**Table 2:** Components of *Yijung-tang* (YJT), *Pyeongwi-san* (PWS), and *Shihosogan-tang* (SST).

Product	Composition (per packet)	Manufacturer
YJT	*Panax ginseng* 2.5 g, *Atractylodes macrocephala* 2.5 g, *dried Zingiber officinale* 2.5 g, *Glycyrrhiza uralensis* 1.25 g	HanKook ShinYak Co., Ltd.
PWS	*Atractylodes macrocephala* 2.5 g, *Citrus unshiu* 1.75 g, *Magnolia officinalis* 1.25 g, *Glycyrrhiza uralensis* 0.75 g, *Zingiber officinale* 0.5 g, *Ziziphus jujuba* 0.67 g	HanKook ShinYak Co., Ltd.
SST	*Bupleurum falcatum* 1.5 g, *Citrus unshiu* 1.5 g, *Cnidium officinale* 1.25 g, *Paeonia lactiflora* 1.25 g, *Poncirus trifoliata* 1.25 g, *Cyperus rotundus* 1.25 g, *Glycyrrhiza uralensis* 0.63 g	Kyungbang ShinYak Co., Ltd.

#### 2.4.2 Criteria for discontinuing or modifying allocated intervention/comparator

Discontinuation or modification of the allocated herbal medicine may occur under the following conditions:

- Development of clinically significant adverse events, including allergic reactions to herbal components or worsening gastrointestinal symptoms judged to be related to the study medication.
- Participant request to stop or change the assigned intervention at any time, without prejudice to subsequent medical care.
- Marked deterioration of functional dyspepsia symptoms requiring additional treatment outside the trial protocol.
- Investigator decision based on medical judgment, protocol non-compliance, or new contraindications (e.g., pregnancy).

Participants discontinuing the allocated intervention will, wherever possible, be encouraged to remain in the trial for outcome assessments to minimize attrition bias.

#### 2.4.3 Concomitant medications and treatments

##### 2.4.3.1 Permitted concomitant medications and treatments

Concomitant medications judged by the principal investigator or sub-investigator to be unlikely to affect the interpretation of study results may be allowed. These may include medications for unrelated conditions or treatments for adverse events that arise during the trial. Whenever possible, the type and dosage of such medications should remain unchanged throughout the study period. All concomitant medications and treatments must be documented in the case report form (CRF), including product name, indication, total daily dose, route of administration, and duration of use. If the dosage of a pre-existing medication is modified or if additional medications are initiated during the trial, this must be promptly reported to the investigator and recorded in the CRF.

##### 2.4.3.2 Prohibited concomitant medications and treatments

The use of new medications or treatments specifically for dyspeptic symptoms is prohibited during the study. If such use occurs, the principal investigator will determine whether the participant should be withdrawn from the trial or excluded from the statistical analysis. The use of antibiotics is strictly prohibited during the study period. Probiotic preparations may be permitted if participants have been taking them continuously for at least 1 month before screening and if the dosage and regimen remain unchanged during the trial. Any change in dosage or regimen during the trial will be considered a protocol violation, and the participant will be withdrawn from the study.

#### 2.4.4 Strategies to improve and monitor adherence

At the baseline visit, participants will receive detailed education on the prescribed dosage regimen and the importance of adherence to the study protocol. To enhance adherence, participants will receive reminder calls during the telephone visits (weeks 2 and 6).

Adherence will be monitored at visits 4 (week 4) and 6 (week 8). Participants are required to return all unused medication packets. Adherence will be calculated as the percentage of packets actually taken relative to the number of packets prescribed: (Total packets dispensed - Total packets returned) / (Total packets scheduled to be taken) × 100%. Participants with less than 70% adherence may be excluded from the per-protocol analysis, and reasons for non-adherence will be documented.

### 2.5 Outcomes and data collection

All instruments used for primary and secondary efficacy endpoints have been previously validated with established reliability and validity in the target population, as detailed below.

#### 2.5.1 Primary efficacy endpoint: GIS score

The GIS score assesses 10 gastrointestinal symptoms: nausea, retching, vomiting, bloating, abdominal cramping/discomfort, early satiety, acid reflux/heartburn, loss of appetite, retrosternal discomfort, and epigastric or upper abdominal pain. Each symptom will be rated on a 5-point Likert scale (0–4), and the total score will be calculated by summing the individual scores (20).

#### 2.5.2 Secondary efficacy endpoints

##### 2.5.2.1 Total dyspepsia symptoms

TDS consists of eight items related to dyspeptic symptoms, including postprandial fullness, bloating, early satiety, epigastric pain, epigastric burning, nausea, vomiting, belching, and other dyspepsia-related symptoms. Each item will be rated on a 4-point Likert scale (0 = none, 1 = mild, 2 = moderate, 3 = severe). The total score, obtained by summing the item scores, indicates the severity of dyspepsia, with higher scores reflecting more severe symptoms (16).

##### 2.5.2.2 Adequate relief for dyspepsia

AR is a commonly used measure in FD evaluations. Participants will be asked whether their dyspepsia-related pain or discomfort has been adequately relieved since the last treatment, responding “yes” or “no.” Responders will be defined as participants answering “yes” to the AR question in at least 50% of assessments over the 8-week trial period. The responder rate will be compared between groups (21).

##### 2.5.2.3 Overall treatment effect

The OTE scale will evaluate participants’ perceived overall improvement in dyspeptic symptoms and quality of life during the clinical trial. Participants will rate their improvement on a 15-point scale ranging from “extremely worsened” to “no change” to “completely improved” (22).

##### 2.5.2.4 Dyspepsia VAS

Participants will assess their subjective dyspepsia severity over the past 2 weeks using a horizontal VAS marked from 0 to 100, where 0 indicates no discomfort and 100 indicates the worst imaginable discomfort. Participants scoring below 40 at screening will be excluded.

##### 2.5.2.5 Functional dyspepsia-related quality of life

The FD-QoL questionnaire will assess the quality of life in dyspeptic patients across four domains: eating, vitality, emotions, and social functioning. It consists of 21 items rated on a 5-point Likert scale, with higher scores indicating better quality of life. Total and domain-specific scores will be calculated (23).

##### 2.5.2.6 Spleen qi deficiency questionnaire

This questionnaire, which assesses appetite, food intake, and general fatigue, consists of 11 items rated on a 5-point scale (0–4). Participants will self-report on nine items, while two items (tongue and pulse diagnosis) will be assessed by a Korean medicine practitioner (24). Scores will be weighted and summed, then divided by four to obtain a total score. Scores exceeding the optimal cutoff of 43.18 will indicate Spleen Qi Deficiency (25).

##### 2.5.2.7 Stomach qi deficiency questionnaire

This questionnaire includes 12 items related to inherent, dietary, and deficiency factors, rated on a 5-point scale (0–4). Participants will self-report on nine items, while three items (abdominal, tongue, and pulse diagnosis) will be assessed by a practitioner. The total score will be calculated, with scores exceeding 14 indicating Stomach Qi Deficiency (26).

##### 2.5.2.8 Food retention questionnaire

This 17-item questionnaire will assess the presence of food stagnation using a 7-point Likert scale (1–7). Scores exceeding 51 will indicate food stagnation in FD patients (27).

##### 2.5.2.9 Cold–heat pattern questionnaire

This self-report questionnaire will include eight items for Cold Syndrome and seven items for Heat Syndrome, rated on a 5-point scale based on typical symptoms over the past 6 months. Scores will be used to classify participants as having Cold Syndrome, Heat Syndrome, Mixed Cold–Heat Syndrome, or Absence of Cold–Heat Syndrome (28).

##### 2.5.2.10 Deficiency–excess pattern questionnaire

Developed through four rounds of Delphi surveys, this questionnaire will include 11 items related to deficiency and excess, with seven items for Deficiency Syndrome and four items for Excess Syndrome. Scores for each item will be summed to calculate deficiency and excess scores (29).

##### 2.5.2.11 Standard tool for pattern identification of FD

Developed based on literature reviews and expert consensus, this 36-item tool will assess participants’ health status over the past 2 weeks in relation to functional dyspepsia. Scores will be weighted to classify participants into six pattern types: spleen and stomach deficiency and cold, dampness and heat in the spleen and stomach, tangled cold and heat, spleen deficiency with qi stagnation, liver–stomach disharmony, and food retention disorder (11).

#### 2.5.3 Safety evaluation variables

Liver function tests (AST, ALT) and renal function tests [blood urea nitrogen (BUN), creatinine (Cr)] will be conducted at screening and at week 8, requiring participants to fast for at least 8 h beforehand. Vital signs, including blood pressure (systolic and diastolic), pulse rate, and body temperature, will be measured. Blood pressure and pulse rate will be recorded in a seated position using an electronic sphygmomanometer after a minimum of 3 min of rest, except during telephone visits. Body temperature will be measured with an electronic thermometer after blood pressure measurement. Adverse events will be collected through participant self-reporting or investigator observation during the clinical trial.

#### 2.5.4 Exploratory evaluation variables

Korean medicine diagnostic devices, including an HRV analyzer, tongue diagnosis device, pulse analyzer, and abdominal examination device, will be used. HRV is a non-invasive tool for assessing health status by measuring sympathetic and parasympathetic activity. Kyung Hee University Hospital at Gangdong will use the SA-6000 (MEDICORE Co., Ltd., Republic of Korea), while Dongguk University Ilsan Oriental Medical Hospital will use the SA-3000NEW (MEDICORE Co., Ltd., Republic of Korea). The tongue diagnosis system (CTS-2000, Daeseong Medical Device, Republic of Korea) will evaluate tongue coating thickness, distribution, and color. The pulse analyzer (DMP-Lifeplus, Daeyo Medi Co., Ltd., Republic of Korea) will provide cardiovascular and pulse wave information. The abdominal examination device (Digital Algometer FPX25, Wagner Instruments, United States) will assess tenderness at nine abdominal acupoints: CV-14, CV-12, left/right ST-20, left/right ST-25, CV-4, and left/right ST-28. Stool and saliva samples will be collected at baseline (week 0) and post-treatment (week 8) for gut microbiome and fecal metabolite profiling and for oral microbiome analysis, respectively.

### 2.6 Data management

All study data will be collected by trained research staff and entered into a secure, password-protected electronic Case Report Form (eCRF) system developed for this trial. The eCRF system includes built-in logic checks and range validations to minimize data entry errors at the point of entry.

Data quality will be regularly monitored by the data management team. Source data verification will be performed by monitors. Queries will be issued for any missing, inconsistent, or outlying data points, and resolved by the site investigators. An electronic audit trail will be maintained to record all changes made to the database.

Participant confidentiality will be strictly maintained. Data will be pseudonymized, with identifying information stored separately and securely on institutional servers, accessible only to authorized personnel. The database will be locked after data cleaning and verification are complete, prior to statistical analysis.

### 2.7 Sample size calculation

Participants will be randomly assigned to either the ACCORD group, which will receive YJT, PWS, or SST based on the machine learning–driven prediction, or the DISCORD group, which will not use the machine learning-based prediction for herbal assignments. Based on preliminary clinical trial results (19), the mean change in the GIS score after 8 weeks in both the ACCORD and DISCORD groups is assumed to be 2.72, with a standard deviation of 4.57. To meet this requirement, each group will require 45 participants. Considering a 10% dropout rate, the total required sample size is 100 participants. This sample size was determined for the prespecified primary comparison (ACCORD vs. pooled DISCORD) and was not inflated to power subgroup or stratified analyses, which will be considered exploratory.

### 2.8 Statistical analysis

All statistical analyses will be conducted using two-sided tests with a significance level of 5%. For continuous data, means and 95% confidence intervals will be presented, whereas categorical data will be presented as frequencies and percentages. All analyses will be performed using SAS® Version 9.4 (SAS Institute Inc., Cary, NC, United States).

#### 2.8.1 Demographic, sociological data, and other pre-treatment participant characteristics

Summary statistics for demographic and sociological variables, as well as pre-treatment participant characteristics, will be presented for each group. Depending on the normality of the data distribution, continuous variables will be analyzed using either the paired t-test or the Wilcoxon signed-rank test, whereas categorical variables will be analyzed using the chi-square test or Fisher’s exact test. If baseline characteristics show statistically significant differences between groups despite randomization, these variables will be included as covariates in the Mixed Model for Repeated Measures (MMRM) for the primary efficacy analysis to statistically adjust for potential confounding effects.

#### 2.8.2 Statistical analysis and evaluation of efficacy endpoints

The primary efficacy endpoint is the between-group difference in the GIS score at week 8 (ACCORD vs. DISCORD). The primary endpoint will be analyzed using the MMRM, with treatment group and visit time as fixed factors and participant as a random factor.

The secondary efficacy endpoints include TDS, AR for dyspepsia, OTE, dyspepsia VAS, FD-QoL, Spleen Qi Deficiency Questionnaire, Stomach Qi Deficiency Questionnaire, Food Retention Questionnaire, Cold–Heat Pattern Questionnaire, and Deficiency–Excess Pattern Questionnaire. Categorical secondary variables will be analyzed using the chi-square test or Fisher’s exact test.

To address the issue of multiple comparisons for the multiple secondary efficacy endpoints, the Benjamini-Hochberg procedure (False Discovery Rate, FDR) will be applied to adjust the *p*-values. The primary endpoint will be tested at a significance level of 0.05 without adjustment. Exploratory outcomes will not be adjusted for multiplicity, and their results will be interpreted as hypothesis-generating.

Exploratory subgroup analyses (by assigned herbal formula and by DISCORD subgroup) will be performed to assess the consistency of treatment effects; these analyses are hypothesis-generating and are not powered for confirmatory inference. Effect modification will be explored using treatment-by-subgroup interaction terms within the mixed model, and results will be reported as effect estimates with 95% confidence intervals and interpreted cautiously.

For the primary outcome, missing data will be handled using the last observation carried forward (LOCF) method. In addition, multiple imputation will be applied as a sensitivity analysis to assess the robustness of results under different assumptions regarding missingness. The main analyses will be conducted on an intention-to-treat basis, with per-protocol analyses performed for comparison.

#### 2.8.3 Safety variable analysis

Safety evaluations will assess the frequency of adverse events suspected to be treatment-related, as well as serious adverse events. Adverse events will be collected through patient self-reports and investigator observations. All observed adverse events will be described in detail, with frequencies of events related and unrelated to the trial intervention presented using descriptive statistics. Group comparisons of adverse events related to medication administration will be conducted using the chi-square test or Fisher’s exact test. Additionally, safety evaluation variables, including AST, ALT, BUN, Cr, and vital signs (blood pressure, pulse rate, and temperature), will be analyzed for pre–post treatment differences using the paired t-test or the Wilcoxon signed-rank test.

### 2.9 Adverse event reporting

#### 2.9.1 Adverse events and serious adverse events

Adverse events (AEs) are defined as undesirable and unintended signs, symptoms, or diseases that occur in participants during a clinical trial, regardless of whether they are causally related to the investigational treatment. AEs are identified through vital sign measurements, participant self-reports, investigator observations, and AE monitoring. Any clinically significant symptoms or changes, as determined by the investigator, are considered AEs and must be documented in the case report form, including details of occurrence, evaluation, and management. Medical events that occur before the administration of the investigational product are documented as medical history, while AEs occurring after trial completion are reported only if they are classified as serious and deemed related to the investigational product.

Serious adverse events (SAEs) are defined as any AE occurring at any dose of the investigational product that results in one or more of the following: (1) death or life-threatening events, (2) hospitalization or prolonged hospitalization, (3) permanent or significant disability/incapacity, (4) congenital anomaly or birth defect, or (5) other medically important conditions, such as drug dependence or abuse, blood disorders, or other significant medical events.

#### 2.9.2 Predictable adverse events and precautions

Adverse reactions that cannot be excluded as causally related to the investigational intervention are considered predictable AEs. In a previous study (30), patients with FD received either Western medicine alone or in combination with YJT for 4 weeks. The combination group reported diarrhea (2 cases), abdominal pain (3 cases), dizziness (1 case), and headache (1 case). In the Western medicine group, diarrhea (2 cases), abdominal pain (1 case), dizziness (1 case), and headache (1 case) were reported. There was no statistically significant difference in AE incidence between groups, and no SAEs were reported in either group. Furthermore, an eight-week clinical trial of modified PWS reported no treatment-related AEs (31). According to clinical practice guidelines for FD (10), six randomized controlled trials reported fewer AEs with SST compared to prokinetic agents (RR 0.32, 95% CI 0.14–0.78). Possible AEs in the present trial include bruising or subcutaneous bleeding from blood draws, rare cases of phlebitis secondary to infection, and transient discomfort or pain associated with abdominal pressure.

### 2.10 Monitoring

Monitoring of this clinical study will be conducted to ensure compliance with the IRB-approved protocol, Good Clinical Practice (GCP) guidelines, and applicable regulatory requirements. Monitoring will be performed either through on-site visits to the clinical trial sites or via telephone by monitors designated by the Korea Institute of Oriental Medicine.

A key component of monitoring is Source Data Verification (SDV). Monitors will systematically validate the survey results and other data entered into the eCRF by comparing them against the original source documents (e.g., medical records, original patient-reported outcome records). Any discrepancies will be managed through a formal data query process until resolved.

The monitoring process includes an initiation visit, routine monitoring visits, and a close-out visit. The initiation visit will occur within 3 weeks after enrollment of the first participant. Routine monitoring visits will be scheduled according to the pace of participant recruitment, and the close-out visit will be conducted after completion of the final participant visit.

Given the established safety profile of the investigational products (YJT, PWS, and SST), which are approved and widely used in Korean medicine, an independent Data Monitoring Committee (DMC) will not be constituted. Instead, the Principal Investigators and the study team will be responsible for ongoing safety monitoring. All Serious Adverse Events (SAEs) will be reviewed immediately upon report.

Independent audits may be conducted by regulatory authorities or the sponsor (National Research Foundation of Korea) to ensure GCP compliance, independent of the routine monitoring activities.

### 2.11 Ethical considerations

The study protocol was reviewed and approved by the IRB of Kyung Hee University Hospital at Gangdong on 9 June 2025 (IRB no. KHNMCOH 2025–04–010-001) and by the IRB of Dongguk University Ilsan Oriental Medical Hospital on 10 June 2025 (IRB no. DUIOH 2025–03–004-005). The protocol has been registered on the Clinical Research information Service (CRiS, registration number: KCT0010587) and the Open Science Framework.^1^ All study-related information will be provided to each participant, and written informed consent will be obtained. The study will adhere to the Declaration of Helsinki and GCP guidelines, and be complied with the Standard Protocol Items: Recommendations for Interventional Trials (SPIRIT; Supplementary material 2).

All personal information collected from potential and enrolled participants will be kept strictly confidential. Each participant will be assigned a unique study identification number, and all data will be recorded and analyzed using this code without direct identifiers. Personal identifiers (e.g., name, address, phone number) will be stored separately in a password-protected file accessible only to the principal investigator and authorized research staff. No identifying information will be disclosed in study reports or publications.

Any substantial modifications to the study protocol (e.g., changes in eligibility criteria, outcomes, or analyses) will be documented in a formal amendment. Such amendments must be approved by the IRBs of both institutions before implementation and updated on the clinical trial registries (CRiS and OSF).

Participants will be covered by clinical trial insurance in accordance with local regulations. In the event of any injury resulting directly from participation in the study, appropriate medical care will be provided, and compensation will be offered as determined by the insurance policy and institutional guidelines.

The results of this study, whether positive, negative, or inconclusive, will be disseminated through publication in a peer-reviewed scientific journal and presentations at relevant national or international conferences. Authorship eligibility will follow the guidelines of the International Committee of Medical Journal Editors (ICMJE).

## 3 Discussion

This clinical trial is a randomized, assessor-blind, parallel, open-label, multi-center study designed to evaluate the predictive accuracy of a machine learning-based herbal medicine response prediction algorithm in patients with FD. Participants are prospectively assigned to receive YJT, PWS, or SST either based on the machine learning–driven prediction algorithm or without the guidance. The predictive performance of the algorithm will be assessed over an eight-week treatment period.

The etiology of FD is heterogeneous and multifactorial, involving gastrointestinal motility disorders, visceral hypersensitivity, immune activity, barrier dysfunction, genetic predisposition, and alterations in the gut microbiota (4). Additionally, psychological factors such as anxiety, depression, somatization, and stress have been shown to influence FD (3). These complex and interrelated mechanisms underscore the need for innovative approaches such as AI to enhance the precision and personalization of treatment strategies.

This study represents an important step forward in applying AI to traditional Korean medicine, particularly for the management of FD. By integrating AI into traditional practice, the study aims to bridge the gap between subjective diagnostic methods and objective, data-driven approaches. The ability of AI to analyze complex datasets and predict individual responses to herbal treatments offers a promising means of addressing the multifactorial challenges of FD. The potential of AI to refine treatment strategies and improve patient outcomes underscores its value as a transformative tool in personalized medicine.

Recent studies have highlighted the potential of AI to revolutionize the diagnosis and treatment of FD. Researchers have developed AI models to differentiate FD from healthy conditions using diverse data sources, including medical imaging and neurological responses (32, 33). These findings suggest that AI-assisted diagnostic tools may enhance both the accuracy and feasibility of FD diagnosis. Furthermore, research efforts are underway to apply AI to pattern identification in FD (34). Machine learning techniques are being used to analyze multiple symptoms and signs, enabling the identification of key indicators that distinguish between different FD patterns. Such approaches underscore AI’s role in refining diagnostic criteria and enhancing the reliability of pattern identification tools, thereby contributing to more accurate and individualized treatment strategies for FD.

Despite these promising advancements, current machine learning models applied to FD face several limitations. Such models depend heavily on large and diverse datasets for training; however, these datasets are often difficult to obtain and may not fully capture the heterogeneity of FD across different populations. This limitation raises concerns regarding the generalizability of AI-driven diagnostic tools. Furthermore, many machine learning models are developed under controlled research conditions but may encounter challenges when implemented in real-world clinical practice, where variability in data quality and patient demographics can substantially affect performance. The multifactorial nature of FD—encompassing gastrointestinal motility disorders, visceral hypersensitivity, and psychological influences—also complicates predictive modeling, as machine learning frameworks typically excel in identifying patterns within more straightforward datasets. Additionally, there is a risk of algorithmic bias, whereby AI-based systems may inadvertently prioritize certain symptoms or demographic groups, potentially skewing diagnostic accuracy and treatment outcomes. These challenges underscore the need for continued refinement of machine learning approaches and rigorous validation of AI applications across diverse clinical settings to ensure both efficacy and reliability.

Beyond the algorithmic challenges, the inherent heterogeneity of FD also poses implications for the statistical analysis of this trial. Given the substantial heterogeneity of FD, the present study was powered for the overall primary comparison (ACCORD vs. pooled DISCORD). Therefore, any subgroup findings should be interpreted cautiously due to limited power after sample fragmentation. Future studies specifically designed and powered to evaluate differential effects in clinically relevant subpopulations are warranted to fully address these complexities.

The decision to exclude a physician-based control group is supported by the current clinical consensus and the inherent challenges in traditional diagnostic practices. According to the Korean Medicine Clinical Practice Guideline for Functional Dyspepsia [10], herbal medicine treatment is primarily guided by Pattern Identification. However, the guideline explicitly states that “evidence for selecting standardized tools to diagnose and evaluate Pattern Identification is currently lacking,” highlighting the difficulty in establishing a standardized diagnostic protocol [10]. It further notes that the personalized nature of diagnosis, which depends heavily on individual patient characteristics and clinician experience, poses a challenge to standardization. Consequently, employing ‘physician’s choice’ as a comparator in this validation phase could introduce substantial inter-physician variability, acting as a confounding factor that obscures the assessment of the machine learning algorithm’s intrinsic predictive accuracy. Despite this methodological rationale, we acknowledge that a significant limitation of this study lies in the design of the control group regarding the interpretation of clinical utility. The DISCORD group, which receives a treatment predicted as suboptimal by the algorithm, does not represent the standard of care (i.e., physician’s choice). While this design is appropriate for validating the predictive accuracy of the model, it cannot demonstrate the superiority of the algorithm over current clinical practice. Consequently, the observed effect size may be larger than what would be observed in a pragmatic trial comparing algorithm-guided treatment to physician’s choice. Future studies comparing the algorithm against standard care are required to establish its real-world clinical utility.

In conclusion, this study marks an important advancement in applying AI to traditional Korean medicine, offering a more personalized and effective approach to the management of FD. By continuing to explore the intersection between technological innovation and traditional practices, the scientific rigor and global credibility of Korean medicine can be further strengthened. Future research should expand the applicability of AI-driven strategies across diverse populations and investigate long-term outcomes to fully realize the potential benefits of this innovative approach.

## Data Availability

The Python scripts used for the development and validation of the machine learning algorithm in the preliminary study are publicly available at [https://github.com/omdnam/FD-formula-recommend-protocol]. The de-identified datasets and the statistical analysis code (SAS) for the main clinical trial may be provided upon reasonable request to qualified researchers with appropriate approvals.

## Footnotes

1 OSF, https://osf.io/2ecz8
